# Intersections of Adverse Childhood Experiences, Race and Ethnicity and Asthma Outcomes: Findings from the Behavioral Risk Factor Surveillance System

**DOI:** 10.3390/ijerph17218236

**Published:** 2020-11-07

**Authors:** Tristen Hall, Ronica Rooks, Carol Kaufman

**Affiliations:** 1 Department of Family Medicine, School of Medicine, University of Colorado Anschutz Medical Campus, Aurora, CO 80045, USA; 2 Department of Health and Behavioral Sciences, College of Liberal Arts and Sciences, University of Colorado Denver, Denver, CO 80204, USA; ronica.rooks@ucdenver.edu; 3 Centers for American Indian and Alaska Native Health, Colorado School of Public Health, University of Colorado Anschutz Medical Campus, Aurora, CO 80045, USA; carol.kaufman@cuanschutz.edu

**Keywords:** adverse childhood experiences, asthma, secondary data analysis, logistic regression models, social determinants of health, health inequities

## Abstract

Racial and ethnic minority subpopulations experience a disproportionate burden of asthma and adverse childhood experiences (ACEs). These disparities result from systematic differences in risk exposure, opportunity access, and return on resources, but we know little about how accumulated differentials in ACEs may be associated with adult asthma by racial/ethnic groups. We used Behavioral Risk Factor Surveillance System data (N = 114,015) from 2009 through 2012 and logistic regression to examine the relationship between ACEs and adult asthma using an intersectional lens, investigating potential differences for women and men aged 18 and older across seven racial/ethnic groups. ACEs were significantly related to asthma, adjusting for race/ethnicity and other covariates. Compared to the reference group (Asians), asthma risk was significantly greater for Black/African American, American Indian and Alaska Native (AIAN), White, and multiracial respondents. In sex-stratified interactional models, ACEs were significantly related to asthma among women. The relationship between ACEs and asthma was significantly weaker for Black/African American and AIAN women compared to the reference group (Asian women). The findings merit attention for the prevention and early detection of ACEs to mitigate long-term health disparities, supporting standardized screening and referrals in clinical settings, evidence-based prevention in communities, and the exploration of strategies to buffer the influence of adversities in health.

## 1. Introduction

Asthma is one of many chronic diseases disproportionately affecting women and racial and ethnic minorities in the United States (U.S.). The prevalence of childhood and adult asthma in the U.S. has been increasing since the 1980s, reaching approximately 8% among both children and adults in 2017 [1,2]. A wide variety of social and environmental risk factors for the development and severity of asthma unfold from early life through adulthood [3,4]. This variation is not randomly distributed through the population, but it reflects differential structures of available opportunities and risk exposures across subpopulations [4]. This distribution does not occur independently by race, ethnicity, sex, or even by severity of cumulated life experiences. Instead, asthma is likely patterned jointly across these characteristics; a unitary focus on just one characteristic will provide incomplete information, likely to the detriment of the most vulnerable. Yet to date, we know little about how accumulated experiences over time might shape the distribution of asthma across race, ethnicity and sex. We draw on intersectionality theory to assess the patterns of asthma burden across the population, examining the relationship between adverse childhood experiences (ACEs) and asthma, and investigating the intersectional contributions of race/ethnicity and sex to shaping that relationship.

### 1.1. The Disproportionate Burden of Asthma

Asthma prevalence, complications, morbidity and mortality are greatest among racial/ethnic minority children and adults [5,6]. Asthma prevalence is greater among women than men (10% vs. 5%) and highest among multiracial, Black/African American, and American Indian and Alaska Native (AIAN) subpopulations, with rates of adult asthma ranging from 7.7% to 8.9%, compared to less than 5% among Asians [7]. Chronic lower respiratory diseases, including asthma, are among the ten leading causes of death for many racial/ethnic minority groups [8]. The contribution of specific risk factors to disparate asthma-related outcomes will facilitate the addressing of root causes and identifying strategies to achieve equitable health outcomes.

### 1.2. Social Determinants of Asthma

Stressors over the life course play a critical role in the development of asthma [9] and have the potential to influence multiple chronic conditions into adulthood, such as heart disease, diabetes and asthma [10]. Social determinants of health are conditions that affect health and life outcomes in the places that people live, learn, work, and play [11]. Childhood may include a variety of influential social determinants of health. Risk factors with the potential for adverse health outcomes include lack of social relationships, poor emotional support, lower socioeconomic status, poor nutrition, and parental stress. Such childhood adversities can foster negative health behaviors, damage the brain, and stunt other physical and psychological development [3]. The accumulation of these adversities throughout one’s lifetime negatively impacts adult health [4]. The synergies (or interruptions) of the social determinants of health and childhood adversities may be particularly salient for the case of asthma. While certainly the relationships of indoor and outdoor pollutants and allergen exposure in early life with asthma development are well-established [12,13], relationships between early life adversities and asthma are less well understood. Childhood adversities are associated with higher adult asthma prevalence [14] and may also partially explain racial/ethnic health disparities in asthma in adulthood [15]. More specifically, stress resulting from lack of social support and exposure to violence combined with mental health conditions like anxiety and depression may contribute to higher rates of asthma in neighborhoods overburdened with these risk factors. These risk factors also happen to be overrepresented in neighborhoods with high proportions of racial and ethnic minority groups [16]. In turn, asthma difficulties can lead to lost school and work days, emergency room visits, hospitalizations, and premature death, with economic and health burdens to patients, their families, and society [1,5]. Evidence linking adult asthma disparities to childhood adversity in the context of vulnerabilities for various social groups may provide a powerful tool to inform interventions at family, clinical, and systems levels in early life. 

### 1.3. Adverse Childhood Experiences as Social Determinants of Health

Children spend much of their time in the home or with family members prior to reaching school age, and household conditions and family dynamics are strongly influential social determinants of health. ACEs, which are traumatic events that occur up to the age of 18, are one measure of home and family conditions. ACEs are defined as verbal, physical, or sexual abuse, as well as family dysfunction [17]. Each additional item endorsed on the ACEs questionnaire contributes to a growing health disadvantage over time. As ACEs build, so does the risk of asthma [14]. The pattern of ACEs varies by race/ethnicity. Black/African American, Hispanic or Latino, and AIAN children experience more adverse events than other racial/ethnic groups [18]. Compared to those who have experienced no ACEs, individuals who have experienced four or more ACEs have a greater risk of numerous health behaviors and outcomes, including unhealthy substance use, sexual risk-taking, poor self-rated health, mental illness, respiratory disease, cardiovascular disease, diabetes, cancer and premature mortality [3,19]. For example, population-based studies in the U.S. demonstrate relationships between ACEs and smoking, which can increase the risk of respiratory conditions including asthma, chronic obstructive pulmonary disease, and lung cancer [15,20]. However, protective resources, such as positive relationships with parents and caregivers, higher socioeconomic status, and safe neighborhoods, can counter the impact of ACEs in childhood and into adulthood. 

Disparities in the prevalence and severity of asthma across race/ethnicity and socioeconomic status reflect differential exposure to adverse social and environmental influences on health. These influences include adverse events, such as insufficient social support, poor family relationships, neighborhood problems, environmental pollution, and financial and social hardships, that can contribute to chronic psychological stress and inflammatory immune processes [16,21,22]. Evidence suggests that the effect of ACEs on health is related to race/ethnicity, sex, and socioeconomic status. However, these relationships are unlikely to be unitary. The consequences of ACEs are not experienced as solely a Black/African American or Asian person, as a man or woman, or as a less educated or lower income person, but patterned by these multiple social statuses together—as a well-educated Black/African American woman, a low-income Asian American man, and so on. 

### 1.4. Adverse Experiences and Asthma: The Intersection of Race/Ethnicity and Sex 

Asthma’s relationship with ACEs and other risk factors likely differs across population groups. Social disadvantages and psychological stressors are cumulative in nature. Certain social groups, particularly those with low socioeconomic status and members of racial and ethnic minority populations, are more likely than others to experience these risk factors, leading to poor health and premature mortality [4]. Additionally, social, financial, and other material resources do not produce the same returns on health for Black/African American individuals compared to White individuals, possibly due to these cumulative effects [23]. Intersectionality theory was developed to address the multiplicative influence of intersecting social identities such as race/ethnicity, sex, and other factors, and how they are related to systems of power, oppression, and various outcomes [24,25,26]. The need for an intersectional lens is demonstrated by stark differences in adverse health outcomes by sex and race/ethnicity. Asthma affects more boys than girls in childhood, but it is more likely to affect women than men in adulthood [5]. Similarly, asthma is not only more prevalent among Blacks/African Americans and Hispanics/Latinos compared to other groups, but more likely to result in morbidity and mortality [27]. Intersectional theory, combined with evidence of the negative health impacts of multiple social determinants of health, suggests that the relationship between ACEs and negative health outcomes may be stronger for racial and ethnic minority populations than others, even after controlling for level of education, socioeconomic status and other key contributors to health outcomes. The combination of inequitable resource distribution and the multitude of environmental, social, and psychological risks to which minority social groups are disproportionately exposed may mean that certain social determinants have a greater impact on the health of members of racial and ethnic minority populations than other groups. Using an intersectional perspective provides a framework to theoretically and methodologically outline the relationship between health disparities and a convergence of identities and experiences that are not often considered jointly. Our approach lays the foundation for appropriately reshaping and redirecting systems of power to those most in need. 

Little empirical research testing the health consequences predicted by intersectionality theory exists [28]. Further, few studies examine how the relationships between ACEs and adverse health outcomes might differ by race/ethnicity and sex, or have sufficient sample sizes and variation to describe outcomes for numerous racial/ethnic subpopulations. In this paper, we address these gaps by modeling intersections between ACEs, race/ethnicity, and sex related to asthma. We hypothesize that:

1. ACE scores have a positively graded relationship with asthma;
2. The positive relationship between ACEs and asthma varies by race/ethnicity, with a stronger relationship experienced by racial/ethnic minority groups with higher rates of asthma and ACEs, such as Black/African American, Hispanic/Latino, AIAN, and multiracial individuals;
3. The positive relationship between ACEs and asthma varies by race/ethnicity and sex, with a disproportionate burden on Black/African American, Hispanic/Latino, AIAN, and multiracial women.

## 2. Materials and Methods 

We conducted secondary data analysis of public surveillance data to assess the relationships between ACEs, race/ethnicity, sex, and adult asthma outcomes. To model intersectionality, we first examined if the relationship between ACEs and asthma varies by race/ethnicity. We then examined whether the relationship between ACEs and asthma varied by race/ethnicity for men and women, respectively. We controlled for education and income in our models, as the literature identifies a consistent inverse relationship between socioeconomic status and asthma [22]. We used the Behavioral Risk Factor Surveillance System (BRFSS), which has a large, nationally representative sample. The sample was large enough to examine the relationship between ACEs and asthma in multiple racial/ethnic groups, with additional interaction models by sex. 

### 2.1. Data Source

This study examined data compiled from the 2009–2012 BRFSS, an annual cross-sectional telephone survey (landline and cell phone), supported by the Centers for Disease Control and Prevention (CDC) and conducted by states. The survey gathered information on U.S. residents’ health behaviors, chronic disease, and preventive care, with more than 400,000 total respondents per year. In addition to core sections that included health status, health care access, chronic health conditions, tobacco use, and demographics, the BRFSS included optional modules which rotated by year, including ACEs [29]. To obtain sufficient numbers for the analysis of small racial/ethnic groups, we aggregated four years of data between 2009 and 2012. A total of 14 states or districts (Arkansas, District of Columbia, Hawaii, Iowa, Louisiana, Minnesota, Montana, Nevada, North Carolina, Oklahoma, Tennessee, Vermont, Washington, and Wisconsin) employed the optional ACEs module in at least one year [30]. The median state-level response rate from 2009 to 2012 BRFSS administrations was about 50% [31]. The complex sampling and weighting methodology of BRFSS is well-documented elsewhere [32]. States that collected optional ACEs modules using their own resources were not required to report resulting data back to CDC for inclusion in public datasets, and national-level datasets between 2013 and 2018 did not include ACEs modules, making 2012 the last available year of relevant data for this study [31].

Combining datasets from 2009 through 2012 for states and districts that utilized the optional ACEs module in any year resulted in a total of 117,876 cases. Of these, 97% had no missing data for variables utilized in this study, 1% were missing only race/ethnicity, and less than 1% had some other combination of one or more missing values for asthma, race/ethnicity, age, education, and smoking history. The exception to this pattern was income, which 13% of respondents declined to provide. Due to the very low prevalence of missing data on the vast majority of variables, we used the original survey dataset rather than imputing values for any variables. This resulted in 114,015 cases for analysis.

### 2.2. Study Variables 

#### 2.2.1. Outcome Variable

Adult asthma prevalence was measured using the questions, “Have you ever been told by a doctor, nurse, or other health professional that you had asthma?” and “Do you still have asthma?” (Yes/No). We categorized all cases responding affirmatively to both questions as having adult asthma.

#### 2.2.2. Exposure Variable 

**Figure 1:**
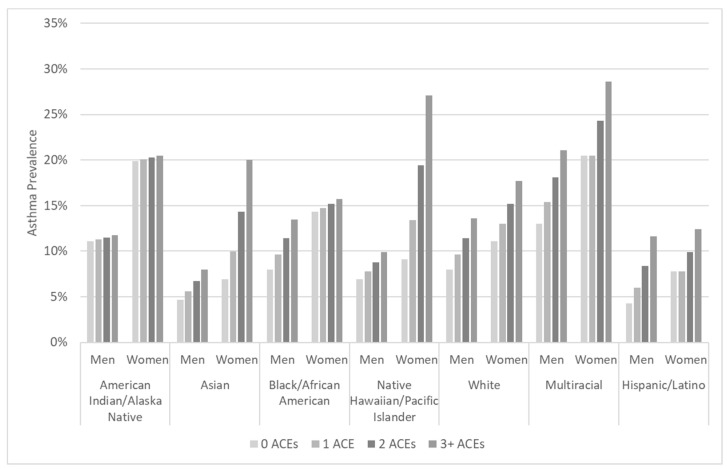
Asthma prevalence estimates using sex-stratified logistic regression for the interaction between race/ethnicity and number of adverse childhood experiences (0–3+), adjusted for age, education, income, smoking history, and geographic region, calculated using Behavioral Risk Factor Surveillance System 2009–2012 data.

ACEs were measured using eleven questions asking respondents whether specific events happened during their childhood, defined as the time period up to age 18. (See Table 1 for survey questions measuring ACEs and corresponding response options.) Some are measured using only response options of “Yes” and “No,” while others take into account the frequency with which the event occurred (Never, Once, More than once). For consistency across measurement of all ACEs, we collapsed responses of “Once” and “More than once” in analyses. We used a count of the total different items experienced by respondents to measure the number of ACEs (range 0–11), with higher scores indicating greater exposure. We also created a dichotomous variable for any ACE vs. none, presented in our descriptive analyses, and a categorical ACEs variable, including 0, 1, 2, and 3+ ACEs, in our asthma prevalence estimates using sex-stratified logistic regression for Figure 1. 

#### 2.2.3. Moderators

We used a calculated variable from the original BRFSS datasets to measure racial/ethnic group. To measure race, respondents were asked, “Which one or more of the following would you say is your race?”, with response options including White, Black/African American or African American, Asian, Native Hawaiian or Other Pacific Islander, AIAN, or Other, with space to specify in open text. To measure ethnicity, respondents were asked, “Are you Hispanic or Latino?” (Yes/No). The calculated BRFSS variable for race/ethnicity categorized respondents into seven mutually exclusive groups of Hispanic or Latino or non-Hispanic White, non-Hispanic Black/African American, non-Hispanic Asian, non-Hispanic Native Hawaiian or Other Pacific Islander, non-Hispanic AIAN, or non-Hispanic multiracial. We recognize that this calculated, mutually exclusive variable oversimplifies the socially constructed concepts of race and ethnic identity and overlooks the substantial variation that exists within such groups [33]. However, examining differences in the contribution of adversity to health outcomes across widely-recognized social categories allows for the elucidation of patterns of health outcomes that are maintained along the lines of these social groupings [34]. For this analysis, we used the combined term “race/ethnicity” to examine possible health disparities associated with the social construction of these categories in the U.S., with their privileges and oppressions. We used Asians as the comparison group since they experience the lowest rate of asthma across groups. While non-Hispanic Whites are often treated as the comparison group in analyses examining disparities across racial/ethnic groups, here we wanted to present a reference of the achievable. As such, we opted to treat the group with the lowest burden of asthma, Asians, as the reference group to demonstrate the full range of disparities across groups. 

#### 2.2.4. Control Variables

Analyses controlled for sex, age, education, income, smoking history, and geographic region. Sex was categorized as male or female on the BRFSS item, “Indicate sex of respondent.” We used “sex” to describe differences between men and women because this was how BRFSS measured the topic, while acknowledging that social aspects related to gender that were not considered in this analysis may also influence chronic disease. Age was measured using the open-ended question, “What is your age?” To describe sample characteristics, we present age in categories of working age adults, 18–64 years, and older adults, 65 years and older. We used the continuous age variable in regression analyses. Education was measured using the question, “What is the highest grade or year of school you completed?” Level of education was categorized into three groups: high school diploma/GED or less, some college or technical school, and graduated from college or technical school. Income was measured using the question, “Is your annual household income from all sources—” with response options in varying increments. We categorized income into five groups: less than USD 20,000; USD 20,000 to less than USD 35,000; USD 35,000 to less than USD 75,000; USD 75,000 or more; and missing or refused to provide income level. Income level was missing for 13% of respondents in the subsample. Because income data is likely not missing at random, following Pedersen, et al. (2017), we grouped respondents who did not provide income level into a “missing” category to retain the complete dataset for analysis [35]. Smoking history was measured using the questions, “Have you smoked at least 100 cigarettes in your entire life?” and “Do you now smoke cigarettes every day, some days, or not at all?” We used a calculated variable for smoking history that coded respondents into two mutually exclusive categories of never- or ever-smoker. Geographic region was measured using the four geographic regions of Northeast, Midwest, South and West, according to state, as defined by the U.S. Census Bureau [36]. In addition to other covariates, geographic region was included as a control variable to account for known geographic differences in asthma prevalence [37], severity [38], and variation in the number of cases available in our sample representing each of these geographic areas. 

### 2.3. Data Analysis 

We used Stata v15.1 software (StataCorp LLC, College Station, TX, USA) for all analyses [39]. We calculated adjusted survey estimates using BRFSS weighting and stratification variables for sample design stratification and stratum weight. We ran frequencies for sample characteristics in the ACEs module of the BRFSS. We computed survey-weighted estimates of asthma prevalence and number of ACEs across the entire sample and stratified by race/ethnicity and sex. 

We used logistic regression to model the relationship between ACEs and adult asthma across racial/ethnic groups, accounting for interactions between race/ethnicity and number of ACEs and controlling for sex, age, education, income, smoking history and geographic region. We then calculated corresponding sex-stratified models, to take into account the intersection of race/ethnicity and sex in the relationship between childhood adversity and adult asthma. These models explored whether the burden of chronic disease was further amplified for men or women in particular racial/ethnic groups. Finally, we presented weighted estimates of asthma prevalence across sex-stratified, racial/ethnic groups for zero, one, two, and three or more ACEs, to illustrate the direct relationship between childhood adversity and adult asthma, and demonstrate disparities in the burden of asthma and ACEs. 

## 3. Results

### 3.1. Sample Characteristics

Women made up about 60% of the sample. The majority of respondents were under the age of 65 (68%), with a mean age of 56 years (SD = 16.8). The racial and ethnic composition of the sample was mostly Whites (82%), followed by Blacks/African Americans (8%), Asians (3%), Native Hawaiian and Pacific Islanders (0.3%), AIANs (2%), multiracial (3%), and Hispanics/Latinos (3%). Table 2 presents summary statistics for sample characteristics. According to comparison surveillance data for the closest available timeframe (tests of comparison were not conducted), the sample characteristics differed from the U.S. population as follows: a greater proportion of survey respondents were diagnosed with asthma (sample: 13%, U.S.: 8%), women (sample: 60%, U.S.: 50%), White (sample: 82%, U.S.: 64%), aged 65 years or older (sample: 32%, U.S.: 13%), or ever-smokers (sample: 48%, U.S.: 39%) [40,41,42].

### 3.2. Asthma

Estimates of survey participants with asthma diagnosed by a health professional indicated wide disparities across race/ethnicity and sex (See Table 3). Adult asthma prevalence was 12% (95% CI = 11.9–12.7%). Asthma prevalence was greater among women (14%, 95% CI = 13.5–14.5%) compared to men (10%, 95% CI = 9.6–10.7%). This pattern remained in all racial and ethnic groups after stratification. Across racial/ethnic groups, asthma prevalence was greatest among those identifying as multiracial, with nearly one-quarter (24%, 95% CI = 20.3–27.3%) of multiracial respondents reporting current-day asthma. 

### 3.3. Adverse Childhood Experiences

In survey-weighted estimates, about 60% of U.S. adults reported any ACEs. The mean number of ACEs across the U.S. population was 1.7 (95% CI = 1.7–1.7). ACEs were similarly prevalent among men (mean = 60%, 95% CI = 58.8–60.5%) and women (mean = 61%, 95% CI = 60.0–61.3%), though on average, women (mean = 1.8, 95% CI = 1.8–1.8) reported slightly more than men (mean = 1.5, 95% CI = 1.5–1.6). Within racial/ethnic groups, multiracial individuals had the greatest number (mean = 2.9, 95% CI = 2.7–3.2) and prevalence (75%, 95% CI = 71.5–78.0%) of ACEs. This was followed by AIANs (69%, 95% CI = 65.1–73.3%; mean = 2.4, 95% CI = 2.2–2.6) and Hispanics/Latinos (68%, 95% CI = 65.6–70.6%; mean = 2.1, 95% CI = 1.9–2.2). The prevalence and mean number of ACEs among women were greater than or approximately equal to men within every racial/ethnic group. See Table 3 for survey estimates of summary statistics for asthma and ACEs for the U.S. population as a whole, stratified by sex and race/ethnicity. 

### 3.4. The Intersection of Adverse Childhood Experiences and Race/Ethnicity

Table 4 contains logistic regression models for asthma predicted by number of ACEs and race/ethnicity, controlling for sex, age, education, income, smoking history, and geographic region. Model 1 includes the main effects of ACEs and racial/ethnic groups independent of one another, Model 2 adds an interaction between ACEs and race/ethnicity, and Models 3a and 3b stratify this interactional model by sex. 

#### 3.4.1. Model 1: Main Effects Model

The number of ACEs was significantly and positively related to asthma outcomes in the main effects model, not accounting for possible differences in the strength of relationship across racial/ethnic groups. Each additional ACE was related to 1.12 times greater odds of asthma (OR = 1.12, CI = 1.10–1.13, *p* < 0.001), controlling for race/ethnicity and other covariates. In comparison to the reference group of Asians, the risk of asthma was significantly greater for Black/African American, AIAN, White, and multiracial respondents, controlling for ACEs and other covariates. The relationship between race/ethnicity and asthma was greatest for multiracial individuals, with a 2.5 times greater risk of asthma than Asian people (OR = 2.54, CI = 1.80–3.59, *p* < 0.001), accounting for ACEs. AIANs were nearly twice as likely as Asians to have asthma (OR = 1.91, CI = 1.30–2.81, *p* = 0.001), independent of other covariates. Compared to Asians, asthma likelihood was about 1.5 times greater for both Blacks/African Americans (OR = 1.57, CI = 1.15–2.14, *p* = 0.004) and Whites (OR = 1.47, CI = 1.11–1.96, *p* = 0.008), controlling for ACEs. Men were significantly less likely than women to have asthma, controlling for ACEs, race/ethnicity, and other covariates (OR = 0.73, CI = 0.67–0.78, *p* < 0.001).

#### 3.4.2. Model 2: Interactional Model

Logistic regression models (Table 4) display the results of examining the interaction between racial/ethnic groups and ACEs to predict asthma. The positive relationship between ACES and asthma was significantly weaker for AIAN individuals compared to the reference group, Asians, and there were no significant differences in the strength of this relationship for other racial/ethnic groups. There remained a positive and statistically significant relationship between ACEs and asthma (OR = 1.25, CI = 1.06–1.49, *p* = 0.009) after including interactions between race/ethnicity and ACEs, indicating that among Asians, the odds of asthma were multiplied by 1.25 with the experience of each additional ACE. The interactions between racial/ethnic groups and ACEs were significant only for AIANs (OR = 0.80, CI = 0.67–0.97, *p* = 0.021), indicating that the relationship between ACEs and asthma is significantly weaker for AIAN compared to Asian individuals, but that the strength of this relationship is not significantly different for any other racial/ethnic group. 

#### 3.4.3. Models 3a and 3b: Sex-Stratified Interactional Models

Models 3a and 3b are sex-stratified logistic regression models taking into account potential differences in the relationship between ACEs and asthma by race/ethnicity, controlling for education, income, smoking history, and geographic region. While the positive and significant relationship between ACEs and asthma remained only among women, the strength of ACEs’ relationship to asthma was significantly lower for Black/African American and AIAN women compared to Asian women. Asian women had a 1.3 times greater risk of asthma for each additional ACE (OR = 1.30, CI = 1.05–1.59, *p* = 0.014). The relationship between ACEs and asthma was significantly lower for Black/African American women (OR = 0.80, CI = 0.65–0.99, *p* = 0.041) and AIAN women (OR = 0.78, CI = 0.62–0.98, *p* = 0.034) compared to Asian women. This interaction was not significant among men for any racial/ethnic group. 

To illustrate the disparities that intersect according to race/ethnicity, sex, and cumulative ACEs, we calculated marginal estimates of asthma prevalence. Figure 1 presents the results for men and women in each of the seven racial or ethnic groups examined in this study. The subgroup with the greatest estimated prevalence of asthma was multiracial women with three or more ACEs, at nearly three in ten (28.6%, CI = 22.0–35.2%). One-fifth of AIAN women (20%, CI = 13.6–27.4%) and 16% of Black/African American women with three or more ACEs (CI = 12.8–18.7%) were estimated to have asthma. The lowest estimated asthma prevalence was among Asian males with no ACEs (4.7%, CI = 2.5–6.9%) and Hispanic/Latino males with no ACEs (4.3%, CI = 2.4–6.2%). 

## 4. Discussion

Study hypotheses were partially supported by analysis results. The findings supported Hypothesis 1, that ACE scores have a positively graded relationship with asthma. Hypothesis 1 was supported by the results of the main effects model examining the independent relationship between ACEs, and race/ethnicity and asthma, as the risk of asthma increased significantly with each additional experience, accounting for control variables. Hypothesis 2 stated that the positive relationship between ACEs and asthma varies by race/ethnicity, with a stronger relationship experienced by racial/ethnic minority groups with higher rates of asthma and ACEs–Black/African American, Hispanic/Latino, AIAN, and multiracial individuals. Hypothesis 2 was partially supported by interactional models, with a significantly lower strength of relationship between ACEs and asthma for AIAN compared to the reference group, Asians, and no significant differences for other racial/ethnic groups. Hypothesis 3, stating that the positive relationship between ACEs and asthma varies by race/ethnicity and sex, with a disproportionate burden on Black/African American, Hispanic/Latino, AIAN, and multiracial women, was partially supported by sex-stratified interactional models, as the positive and significant relationship between ACEs and asthma was maintained for women but was no longer significant among men, and the strength of relationship between ACEs and asthma was, unexpectedly, significantly lower for Black/African American and AIAN women compared to Asian women. 

Research has long established socially determined variations in health outcomes by ACEs, race/ethnicity and sex. Our paper contributes substantially to this work by considering the intersection of all three characteristics as related to one outcome, asthma. Our analysis reinforces prior work in this area: the number of ACEs and being female are independently and significantly related to the probability of a reported diagnosis of asthma. Contrary to our hypotheses, the results suggest that there were no significant differences in the relationship between ACEs and asthma for most racial/ethnic groups, with the exception of Black/African American and AIAN women, for whom the relationships between ACEs and asthma were actually weaker than for Asians. The findings allow a greater understanding of how various social identities and early life experiences interact with one another in relation to asthma likelihood, and provide insight for prioritizing potential strategies for asthma prevention. 

Our findings show that negative social and environmental determinants of health expand health inequities across disadvantaged social groups and the general population. The odds of asthma increase significantly with each added ACE, independent of other factors related to race/ethnicity and other covariates. AIAN, Black/African American, White, and multiracial individuals experience a significantly greater risk of asthma than Asians related to their race/ethnicity, over and above ACEs and covariates. Additionally, AIAN and Black/African American women experience a significantly weaker relationship between ACEs and asthma compared to Asian women, while among men there was no significant relationship between ACEs and asthma, nor differences in the strength of relationship between ACEs and asthma across race/ethnicity. The findings suggest that women are particularly vulnerable to long-term asthma outcomes related to ACEs. This may contribute to higher rates of adult asthma for women compared to men. This merits the paying of increased attention to the prevention and early detection of ACEs for women in particular so as to mitigate long term health disparities. 

While intersectionality theory would suggest the multiplicative effects of structural inequalities, an intersectional relationship was not fully supported for all racial/ethnic groups. The lack of significant interaction between most racial/ethnic groups and ACEs may lend some support to a multiple jeopardy hypothesis in which the effects of membership in such groups are instead additive. While social identities are interdependent on one another, results suggest that the relationship between ACEs and adult asthma differs in strength only between Asians, Blacks/African Americans, and AI/ANs. This also highlights the challenges and limitations inherent to using statistical methods, including interaction terms, to model intersectionality [43].

Our findings point to childhood circumstances and sex as multiple intersecting identities that are important to wellbeing. However, they say little about causal mechanisms. We know little about what components at the intersection of experiences and social identity propel this vulnerability for some and not others. One potential explanation, in line with intersectionality, is that perceptions of adversity or the definitions of specific ACE survey items may differ across racial/ethnic groups, possibly intersecting with sex. Measures of childhood adversity may need to be expanded to include additional experiences, such as food insecurity, homelessness, or the prolonged absence of a parent [18], or adversity occurring outside of the home, such as experiencing racism, bullying, or violence [44]. For some AIAN groups, social support and community belonging may act as protective factors against the influence of ACEs on health outcomes [45,46]. This could explain the observed significantly lower likelihood of asthma with respect to ACEs for AIAN women. It is also possible that other factors that influence asthma outcomes are more relevant than ACEs for some groups, such as neighborhood exposure to negative environmental impacts [47], poor psychological wellbeing, and a family history of asthma [48,49]. Another potential contributor could be that some groups spend more time in housing or work spaces that are constructed with toxic materials that heighten vulnerability to asthma [47,50], which would be reflected in higher asthma rates but not the analyses presented in this study. Further research in this area could illuminate how specifically resources and supports may act as a buffer against negative health outcomes, or how influences other than ACEs more strongly affect asthma outcomes for some groups.

Interventions targeting asthma or ACEs can be better informed by focusing on multiple vulnerable statuses to reduce health disparities. Effective evidence-based interventions for the primary prevention of asthma do not yet exist [51], but interventions to reduce exposure to environmental triggers [52,53], increase self-management skills, or incorporate psychological counseling or relaxation strategies are effective for asthma control and management [54,55]. These interventions are most effective when they address the multiple physical and social factors that contribute to asthma, are tailored for participants’ needs and level of risk, and delivered in venues where the target audience can be effectively and widely reached [56]. Such evidence highlights the need for asthma prevention and management interventions that address the social environment, in addition to resources focusing on reducing the exposure to allergens in homes, schools, and outdoors [57]. 

Our findings support ongoing calls for standardized screening and referrals for ACEs and social needs more broadly in clinical settings [58,59,60], as well as evidence-based prevention efforts and interventions in the wider community [61]. One such approach is trauma-informed care, in which healthcare organizations systematically recognize the effects of childhood adversity and offer effective treatment options [62]. Interventions that target those with multiple risk factors for adversity and chronic disease, and select home visitation programs and social work strategies that strengthen peer, family, and community social support, particularly in early childhood years, are effective in the prevention of child abuse and neglect [63,64]. These approaches to preventing or buffering the effect of ACEs could contribute to reductions in asthma disparities across sex and racial/ethnic groups. More broadly, study findings reinforce the need for asthma prevention and treatment interventions that consider social determinants of asthma, such as stress, violence, socioeconomic conditions, and residential segregation, which drive disproportionate medical care access, exposure to pollutants and allergens, and levels of collective efficacy and other social resources [16]. The Empower Action Model is one example of a systematic framework designed to prevent childhood adversity and associated negative health outcomes. This model consists of multilevel approaches that emphasize protective factors, such as developing resilience through interpersonal interventions that promote stress management skills in combination with corresponding policies to build healthy environments [65]. The implementation of such interventions by organizations and communities would be consistent with calls from the CDC for investment in evidence-based strategies that support healthy relationships and supportive environments to prevent chronic disease in adulthood [66]. Future research building on these findings could examine the potential differences in the severity of asthma according to ACEs and racial/ethnic groups so as to further untangle the nature of the relationship between ACEs and asthma across racial/ethnic groups. Additionally, this study sets the foundation for further research exploring the reasons behind unexpected findings for some racial and ethnic minority groups, and the effectiveness of multilevel, intersectional interventions to reduce the burden of ACEs on health. 

### Strengths and Limitations

This study has several strengths. Conventional analytic methods do not perfectly model intersectional health effects [26]. However, our stratified interaction analysis does allow for conclusions about the effect of differential moderation of multiple racial/ethnic groupings on the relationship between ACEs and asthma. This represents some of the first evidence examining the relationship of ACEs with adult asthma by both sex and race/ethnicity—also unique in its examination of several racial/ethnic subpopulations. 

This study also has a number of limitations. This is a cross-sectional study, and conclusions regarding causal relationships between exposures and outcome cannot be made. BRFSS is telephone-based and self-reported, and therefore it may miss U.S. residents without landline or cellular telephone access. Data for one key exposure variable, ACEs, were collected in only a subset of states, which may result in bias. Sample characteristics differed somewhat from U.S. population estimates. The overrepresentation of Whites in the BRFSS sample could conservatively skew estimates for asthma and ACEs, as well as the main effect of ACEs on asthma outcomes. Conversely, the overrepresentation of females may positively skew asthma estimates. The ACEs measurements used do not account for the timing of childhood adverse events, most notably whether experiences happened during particularly sensitive periods. Longitudinal data documenting the specific timing and frequency of adverse events would provide greater insight into cumulative disadvantage as a potential mechanism for the influence of ACEs on asthma. The retrospective measure of ACEs creates the potential for recall bias among participants. Asthma is measured using the self-report of a health professional’s asthma diagnosis, which excludes those who have not been formally diagnosed, potentially underestimating asthma prevalence. For the most part, potential biases would be expected to generate findings that underestimate ACEs and their contribution to asthma. The BRFSS data did not allow for incorporating specific built environmental exposures that may influence asthma, such as air pollution. Finally, the number of respondents in some analysis subgroups resulted in relatively wide confidence intervals, limiting the ability for comparisons.

In spite of these limitations, this study provides insight into the interplay of characteristics that may put individuals at risk of asthma. Our work strongly suggests that the increased attention paid to ACEs in recent years should include an accounting of sex and race/ethnicity to advance health equity. Building upon these findings, future studies might examine the relationships between ACEs, race/ethnicity, age, and other chronic diseases such as cardiovascular disease and type 2 diabetes that disproportionately affect minority populations, and the potential mediating influence of certain health behaviors. 

## 5. Conclusions

We used an intersectional approach to address patterns of asthma across race/ethnicity and sex within the context of childhood adversity experiences. The likelihood of asthma is particularly high for women reporting ACEs as well as individuals identifying as AIAN, Black/African American, multiracial, or White, though there were few differences in the strength of relationship between ACEs and asthma across racial/ethnic groups. Assessing patterns of disease or dysfunction using this approach contributes powerful evidence to prevention strategies prioritizing the influential early childhood period. It supports investment in culturally appropriate prevention and treatment strategies throughout the life course for those most at risk, which may prove to have positive and long-lasting mitigating effects on the substantial burden of chronic disease in the U.S. 

## Figures and Tables

**Table 1:** Survey questions and response options for the adverse childhood experience module of the Behavioral Risk Factor Surveillance System, 2009–2012.

Survey Question	Response Options and Coding for Analysis
1. Did you live with anyone who was depressed, mentally ill, or suicidal?	0—No 1—Yes
2. Did you live with anyone who was a problem drinker or alcoholic?	
3. Did you live with anyone who used illegal street drugs or who abused prescription medications?	
4. Did you live with anyone who served time or was sentenced to serve time in a prison, jail, or other correctional facility?	
5. Were your parents separated or divorced?	
6. How often did your parents or adults in your home ever slap, hit, kick, punch or beat each other up?	0—Never 1—Once 1—More than once
7. Before age 18, how often did a parent or adult in your home ever hit, beat, kick, or physically hurt you in any way? Do not include spanking.	
8. How often did a parent or adult in your home ever swear at you, insult you, or put you down?	
9. How often did anyone at least 5 years older than you or an adult, ever touch you sexually?	
10. How often did anyone at least 5 years older than you or an adult, try to make you touch them sexually?	
11. How often did anyone at least 5 years older than you or an adult, force you to have sex?	

**Table 2:** Sample characteristics, Behavioral Risk Factor Surveillance System 2009 to 2012 adverse childhood experiences module data.

Sample Characteristics	Percentage or Mean (SD)
	*N* = 114,015
Asthma	12.70%
Adverse Childhood Experiences	
Any	57.10%
Number	1.5 (2.0)
Sex	
Male	39.70%
Female	60.30%
Age (years)	55.7 (16.8)
18–64	68.20%
65+	31.80%
Race/Ethnicity	
American Indian/Alaska Native	1.70%
Asian	2.70%
Black/African American	7.60%
Native Hawaiian/Pacific Islander	0.30%
White	81.90%
Multiracial	2.70%
Hispanic	3.20%
Level of Education	
HS/GED or less	36.90%
Some college/tech. school	27.80%
College/tech. school grad	35.20%
Annual Income	
USD <20,000	15.80%
USD 20,000 – <35,000	20.20%
USD 35,000 – <75,000	28.30%
USD 75,000+	23.30%
[Missing/Refused]	12.40%
Smoking History	
Ever Smoker	47.50%
Geographic Region	
Midwest	28.90%
Northeast	11.30%
South	31.70%
West	28.10%

**Table 3:** Survey prevalence estimates (% or mean, 95% Confidence Interval) for asthma and adverse childhood experiences, overall and stratified by sex and race or ethnicity, calculated using Behavioral Risk Factor Surveillance System 2009–2012 data, N = 114,015.

	Asthma			Any ACE			# ACEs		
	All	Men	Women	All	Men	Women	All	Men	Women
Overall	12.3 (11.9–12.7)	10.1 (9.6–10.7)	14.0 (13.5–14.5)	60.2 (59.7–60.7)	59.6 (58.8–60.5)	60.7 (60.0–61.3)	1.7 (1.7–1.7)	1.5 (1.5–1.6)	1.8 (1.8–1.8)
American Indian/Alaska Native	17.6 (14.1–21.0)	12.6 (7.8–17.3)	21.8 (16.8–26.8	69.2 (65.1–73.3)	69.3 (62.7–75.9)	69.1 (63.9–74.3)	2.4 (2.2–2.6)	2.3 (2.0–2.7)	2.5 (2.2–2.8)
Asian	8.2 (6.1–10.3)	5.9 (3.0–8.7)	10.1 (7.1–13.2)	43.7 (39.3–48.1)	43.1 (36.3–50.0)	44.1 (38.5–49.8)	0.9 (0.8–1.0)	0.8 (0.6–1.0)	1.0 (0.8–1.2)
Black/African American	14.2 (12.8–15.5)	11.1 (8.8–13.3)	16.0 (14.2–17.7)	64.8 (63.0–66.5)	64.6 (61.5–67.7)	64.8 (62.7–67.0)	1.9 (1.8–2.0)	1.8 (1.6–1.9)	2.0 (1.8–2.1)
Native Hawaiian/Pacific Islander	13.2 (7.2–19.3)	8.1 (0.8–15.3)	19.9 (10.9–28.8)	57.9 (43.7–72.0)	49.6 (27.2–71.9)	68.6 (56.9–80.3)	1.7 (1.2–2.1)	1.1 (0.6–1.5)	2.4 (1.8–3.0)
White	12.0 (11.6–12.4)	9.9 (9.3–10.5)	13.7 (13.1–14.2)	59.2 (58.6–59.8)	58.7 (57.8–59.6)	59.6 (58.9–60.4)	1.6 (1.6–1.7)	1.5 (1.4–1.5)	1.8 (1.7–1.8)
Multiracial	23.8 (20.3–27.3)	20.1 (15.2–25.0)	27.1 (22.2–32.0)	74.7 (71.5–78.0)	75.4 (70.6–80.2)	74.2 (69.7–78.7)	2.9 (2.7–3.2)	2.8 (2.5–3.1)	3.1 (2.8–3.4)
Hispanic/Latino	9.6 (8.0–11.3)	9.1 (6.7–11.5)	10.2 (7.9–12.4)	68.1 (65.6–70.6)	66.1 (62.3–69.9)	69.9 (66.6–73.3)	2.1 (1.9–2.2)	1.9 (1.7–2.1)	2.3 (2.0–2.4)

**Table 4:** Overall and sex-stratified logistic regression for asthma predicted by number of adverse childhood experiences and race/ethnicity, adjusting for interaction between ACEs and race/ethnicity, covariates, calculated using Behavioral Risk Factor Surveillance System 2009–2012 data.

Predictor Variables	Model 1–Main Effects ^1^		Model 2–Interaction ^1^		Model 3a–Interaction, Men ^1^		Model 3b–Interaction, Women ^1^	
	OR (95% CI)	*p* > |t|	OR (95% CI)	*p* > |t|	OR (95% CI)	*p* > |t|	OR (95% CI)	*p* > |t|
# ACEs	1.12 (1.10–1.13)	<0.001 *	1.25 (1.06–1.49)	0.009 *	1.11 (0.93–1.32)	0.25	1.30 (1.05–1.59)	0.014 *
Race/Ethnicity (Reference = Asian)								
American Indian/Alaska Native	1.91 (1.30–2.81)	0.001 *	2.97 (1.89–4.65)	<0.001 *	2.58 (1.14–5.84)	0.023 *	3.12 (1.85–5.25)	<0.001 *
Black/African American	1.57 (1.15–2.14)	0.004 *	2.06 (1.49–2.85)	<0.001 *	1.80 (0.98–3.32)	0.058	2.07 (1.45–2.95)	<0.001 *
Native Hawaiian/Pacific Islander	1.57 (0.89–2.78)	0.123	1.51 (0.73–3.13)	0.266	1.37 (0.38–5.02)	0.63	1.71 (0.81–3.59)	0.158
White	1.47 (1.11–1.96)	0.008 *	1.69 (1.26–2.26)	<0.001 *	1.77 (1.04–3.01)	0.037 *	1.57 (1.14–2.16)	0.005 *
Multiracial	2.54 (1.80–3.59)	<0.001 *	2.74 (1.79–4.19)	<0.001 *	2.70 (1.28–5.68)	0.009 *	2.61 (1.58–4.30)	<0.001 *
Hispanic/Latino	0.97 (0.68–1.36)	0.843	1.06 (0.72–1.56)	0.774	1.18 (0.62–2.22)	0.619	0.92 (0.57–1.49)	0.739
Interaction: ACEs by Race/Ethnicity								
American Indian/Alaska Native			0.80 (0.67–0.97)	0.021 *	0.89 (0.71–1.12)	0.32	0.78 (0.62–0.98)	0.034 *
Black/African American			0.84 (0.71–1.01)	0.058	1.01 (0.82–1.24)	0.954	0.80 (0.65–0.99)	0.041 *
Native Hawaiian/Pacific Islander			0.96 (0.75–1.23)	0.771	1.03 (0.65–1.63)	0.892	0.92 (0.70–1.21)	0.548
White			0.90 (0.76–1.06)	0.205	1.01 (0.85–1.21)	0.887	0.87 (0.70–1.07)	0.18
Multiracial			0.91 (0.76–1.09)	0.315	1.04 (0.83–1.30)	0.713	0.88 (0.70–1.10)	0.25
Hispanic/Latino			0.91 (0.76–1.09)	0.305	1.04 (0.85–1.27)	0.694	0.88 (0.70–1.10)	0.257
Sex (Reference = Women)								
Men	0.73 (0.67–0.78)	<0.001 *	0.73 (0.67–0.79)	<0.001 *	--	--	--	--
Constant	0.13 (0.10–0.19)		0.12 (0.08–0.16)	<0.001 *	0.08 (0.05–0.15)	<0.001 *	0.12 (0.08–0.17)	<0.001 *

^1^ All models control for education, income, age, smoking history, and geographic region (not shown); * *p* < 0.05.
